# Relative, individualized, or normalized? A conceptual clarification of running speed terminology in soccer

**DOI:** 10.3389/fspor.2026.1873257

**Published:** 2026-07-29

**Authors:** Ricardo Pimenta, Hugo Antunes, José Afonso, Fábio Yuzo Nakamura

**Affiliations:** 1 Research Center in Sports Sciences, Health Sciences and Human Development, CIDESD, University of Maia, Maia, Portugal; 2 Research Center of the Polytechnic Institute of Maia (N2i), Maia Polytechnic Institute (IPMAIA), Castêlo da Maia, Maia, Portugal; 3 Department of Rehabilitation and Optimization of Performance (DROP), Futebol Clube de Famalicão - Futebol SAD, Famalicão, Portugal; 4 FSI Lab, Football Science Institute, Granada, Spain; 5 Centre for Research, Education, Innovation and Intervention in Sport (CIFI2D), Faculty of Sport of the University of Porto, Porto, Portugal; 6 Graduate Program in Physical Education, Federal University of Pernambuco, Recife, Brazil

**Keywords:** external load, football, normalization, performance analysis, training load

## Abstract

There is inconsistency in quantifying, defining and interpreting running speed nomenclature in soccer. In particular, the terms “relative”, “individualized”, and “normalized” are frequently used interchangeably, despite referring to different conceptual and methodological approaches. This lack of clarity may affect the interpretation of running intensity and limit comparability across studies. Therefore, the aim of this paper is to clarify the conceptual distinctions between these terms in the context of soccer performance analysis. We distinguish between absolute thresholds (e.g., a value expressed without context), relative comparisons based on external references, individualized reference values (e.g., a value obtained during treadmill protocol), and normalized metrics that express running speed as a proportion of an individual's physiological characteristic (e.g., percentage of the maximum speed). Using theoretical arguments and applied examples, it was shown that many approaches described as “relative” or “individualized” correspond, in methodological terms, to normalization. Moreover, it was also highlight that the interpretation of normalized intensity depends on the reference used, as different tests (e.g., linear sprint tests vs. intermittent field tests) reflect distinct physiological and mechanical characteristics. Clarifying these distinctions is important to improve methodological rigor and consistency in the analysis of running demands. By contextualizing running demands according to each athlete's maximum speed registered until the point of monitoring, this approach may better approximate the normalized locomotor stimulus imposed by a given training load, in relation to the player's true, albeit inherently unknown, maximal potential exposure. Future research should adopt more precise terminology and clearly report the reference values and scaling procedures used when quantifying running demands in soccer.

## 1 Introduction

Running performance is one of the most commonly used components of soccer performance analysis. With the development of tracking technologies such as Global Navigation Satellite System (GNSS), Local Positioning System (LPS) and optical systems, it is now possible to quantify external load during training and matches with precision (1, 2). Locomotor variables such as high-speed running, sprinting, accelerations, and decelerations are commonly derived from tracking systems. However, the operational definition of these variables may differ according to the tracking-system manufacturer, the proprietary data-processing algorithms, and the extent to which practitioners retain default settings or customize specific thresholds. In previous studies, tracking-derived locomotor variables have been classified using either predefined absolute thresholds, such as > 19.8 km/h or > 25.2 km/h to define high-speed running or sprinting intensities (3, 4), or thresholds adjusted according to individual player capacities. The latter approaches have been described across studies using different terms, such as “relative” (5–7), “individualized” (8–10) and “normalized” that are often used interchangeably (3–6, 8, 9), when the methodological process being applied is, in fact, a form of normalization (3, 4), which consists of scaling a given locomotor metric to the player's capacity and/or characteristic.

Importantly, the term “individualized” refers to the use of athlete-specific reference values (e.g., MS, MAS, or VO₂max) to define or scale performance metrics, rather than to the mere expression of absolute values *per se*. For example, a player's absolute average speed during a cardiopulmonary test (e.g., 15 km/h) represents an individual performance value but is neither relative nor normalized to a reference value such as the final speed obtained in the cardiopulmonary test. In contrast, when speed is expressed as a percentage of an individual's maximum speed (MS), the procedure involves ratio-based normalization using an individual reference value. In this context, the term “relative” lacks specificity, as it does not distinguish between different underlying mathematical procedures. More broadly, when running speed is mathematically scaled in relation to a biomechanical or physiological parameter (e.g., expressed as a ratio or pecentage to MS), the procedure corresponds to normalization. This distinction is not merely semantic, as it may influence the interpretation of external-load metrics. Clarifying the distinction between relative expressions, individualized reference frameworks, and normalized variables may therefore improve methodological rigor and enhance comparability across studies in performance analysis and training monitoring.

**Figure 1:**
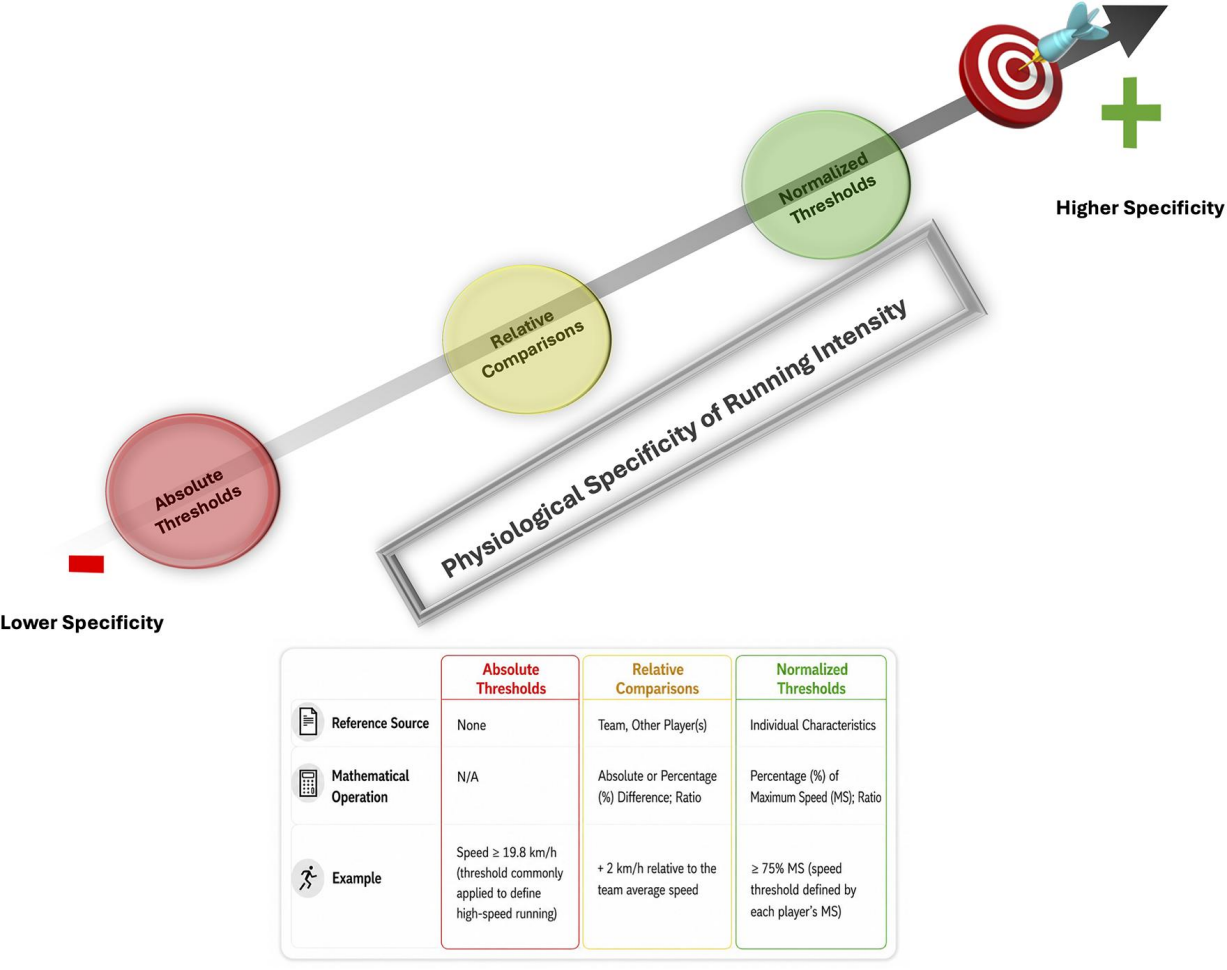
Conceptual progression in the interpretation of running-speed demands in soccer. Absolute thresholds apply fixed speed values across players. Relative comparisons contextualize performance against external references. Normalized thresholds scale running speed as a percentage of an individual physiological characteristic (e.g., % of MS).

According to the methodologies and stated aims of previous studies, the interchangeable use of these terms appears to be associated with the application of the training principle of individualization (Figure 1). In practice, researchers have normalized participants' MS, thereby deriving normalized speed thresholds to characterize the external load experienced by each individual. This may reflect some conceptual overlap between the terms individualization, relativization, and normalization, potentially contributing to terminological ambiguity and, ultimately, to their inappropriate application.

Therefore, the aim of this paper is to clarify the conceptual distinction between “relative”, “individualized”, and “normalized” running speed metrics in soccer performance analysis.

## 2 Interchangeability between individualized and relative expressions of running speed

A term frequently used in soccer performance analysis is “individualized” (8, 9, 11). However, “individualized” primarily identifies the source of the reference value rather than the mathematical operation applied to the variable (11). As such, these three concepts could be distinguished according to the origin of the reference value, the type of comparison, and the scaling procedure used to express running speed. A threshold may be described as individualized since it was derived from a player-specific parameter, but it does not concomitantly indicate that running speed was expressed in absolute terms, as a percentage of a physiological or biomechanical characteristic, or through another normalization procedure.

Similarly, the term “relative” could refer to a range of contextual comparisons. For example, running speed may be interpreted relative to team averages, normative values derived from population datasets, positional benchmarks, or even the performance of an opponent (6, 12, 13). In these cases, the reference value is external to the player's own physiological or biomechanical characteristic, without necessarily applying a scaling procedure to an individual parameter. Although such comparisons may provide useful contextual insights, they do not necessarily reflect the physiological, mechanical, or perceptual intensity experienced by the player.

Distinguishing these concepts accurately is essential given that they are often used interchangeably to describe thresholds derived from player-specific parameters such as MS, MAS or anaerobic speed reserve (ASR). For example, Abt and Lovell (2009) defined individualized high-intensity thresholds by assessing the running speeds associated with the second ventilatory threshold (9). Likewise, Rago et al. (2020) reported individualized speed zones based on a combination of MAS, MS, and ASR (11). Although the use of the terms “individualized” or “relative” is not necessarily incorrect in these examples, the underlying analytical procedure can also be interpreted as a form of normalization, since running speed is scaled according to an individual physiological or biomechanical reference. Similarly, other studies combine both terms, such as “individual relative thresholds', to describe speed or acceleration zones derived from player-specific parameters (5, 14). For instance, although one article was titled “*Use of Individual Relative Thresholds to Assess Acceleration in Young Soccer Players According to Initial Speed*”, the methodological procedure employed in the study describes a normalization. This does not diminish the relevance of the study; rather, it illustrates how terminology used in titles and abstracts may not always fully reflect the underlying analytical procedure.

By contrast absolute running-speed thresholds are typically applied equally to all players, such as defining sprinting as running above a fixed value, for example ≥ 25.2 km/h. The threshold itself is not derived from the individual player's physiological or biomechanical characteristics. Nevertheless, absolute thresholds may still be used to compare intra- or inter-individual performances, although not indicating whether the same external speed represents the same internal or mechanical demand across players. As an example running-speed output may therefore be described as “high relative to the team average”, “above the positional norm”, or “250 m above 25.2 km/h”.

For these reasons, describing speed thresholds derived from MS, MAS or ASR simply as “relative” or “individualized” may be conceptually imprecise when the objective is to interpret external load in relation to a player's specific-parameter MS. A more precise description should specify whether the threshold is absolute, contextually relative, individualized or normalized. It is therefore suggested that authors explicitly report the source of the reference value, the mathematical scaling procedure, and the intended interpretation of the resulting speed zones.

## 3 Normalization to Maximum speed

Normalization refers to the process of scaling a variable according to an individual physiological reference, typically expressed as a proportion or percentage of a maximum value. In the present study, the term normalization is used specifically to describe ratio-based and percentage physiological normalization and not broader statistical normalization procedures such as z-score transformations, allometric scaling, or other standardization methods. Normalization to physiological maximum is widely used across multiple areas of exercise science. Aerobic intensity is commonly normalized to maximal oxygen uptake (%VO₂max) (15), cardiovascular load to percentage of maximal heart rate (%HRmax) (16), and resistance training loads to percentage of one-repetition maximum (%1RM) (17). In biomechanics, mechanical outputs such as joint torque are frequently normalized to body mass (e.g., Nm/kg) or to maximal voluntary contraction to allow meaningful comparisons between individuals with different anthropometric characteristics (18). These normalization procedures allow physiological and mechanical intensities to be expressed as a proportion of maximal or size-related characteristics, rather than as absolute values. Applying the same principle to locomotor speed in soccer allows running intensity to be contextualized relative to each player's MS. Accordingly, the same fraction of MS would correspond to an equivalent normalized locomotor intensity, although not necessarily to an equivalent physiological or metabolic intensity.

Examples of normalized running intensities include values such as 60%, 75% or 90% of MS (3, 4). By expressing speeds as proportions of MS [tested in a 40-m linear sprint test (19)], normalization ensures that the running intensity represents the same fraction of maximum performance potential for every player.

**Figure 2:**
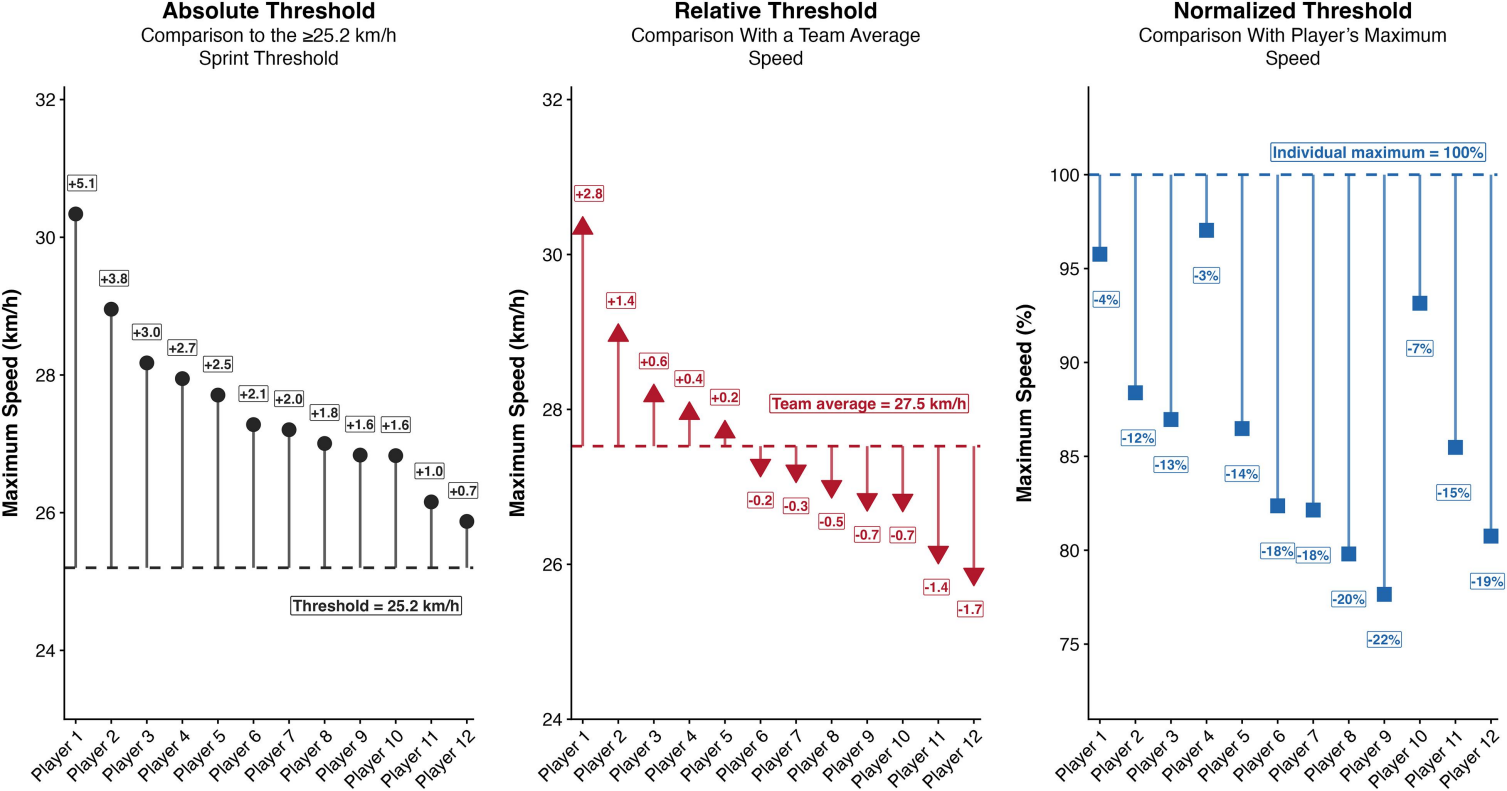
Conceptual comparison of absolute, relative, and normalized running-speed thresholds across players with different MSs. The absolute threshold (left) applies a fixed speed value (e.g., ≥25.2 km/h) equally to all players, regardless of individual characteristic. The relative threshold (middle) expresses speed in relation to an external reference (e.g., group mean MS), allowing contextual comparison but not accounting for individual physiological differences. The normalized threshold (right) scales running speed as a percentage of each player's individual MS (%MS), ensuring that the same threshold represents an equivalent proportion of maximal speed across players.

Full normalization would require expressing locomotor thresholds scaled to each player's MS. Without this reference, players with lower MS (e.g., 30 km/h) would experience substantially different biomechanical patterns, metabolic demands, and locomotor stress at the same externally defined thresholds (Figure 2). Consequently, the physiological and mechanical implications of these speed zones may differ considerably across players.

This distinction highlights an important methodological consideration for locomotor load quantification in team sports: MS thresholds based on an external reference may improve conceptual interpretation compared to purely absolute thresholds, yet they still fail to fully account for inter-individual variability in MS. As previously discussed, the normalization of locomotor thresholds to individual MS may provide a more precise approach for capturing the true intensity of running demands experienced by each player (4).

For example, the study conducted by Amman et al. (2022) demonstrated an important conceptual distinction in the definition of high-speed running and sprinting thresholds. Specifically, the authors expressed the absolute speed thresholds of ≥19.8 km/h and ≥25.2 km/h relative to a reference MS of 36 km/h, which corresponded to 55% and 75% of this value, for high-speed running and sprinting, respectively. This methodological approach enables a clear distinction between absolute and externally referenced thresholds. However, the MS used (36 km/h) was derived from previously reported data rather than from the individual MS of each player (20). Consequently, the thresholds were expressed relative to a standardized reference speed, rather than to each player's MS.

As a result, the thresholds corresponded to 55% and 75% of the reference speed, which indirectly introduces some level of “individual differentiation”, since each player's locomotor profile will interact differently with these thresholds. In practice, because players possess different MS, the relative physiological and biomechanical demands associated with these thresholds will vary between individuals. Therefore, while the thresholds are expressed as percentages, they cannot be considered truly individualized or normalized.

Finally, running speeds have been normalized either to the MS attained during a 40-m linear sprint test (19) or to the MS (more adequately defined as final speed of the test) reached in an intermittent field test (e.g., 30-15_IFT_) (21). Although both approaches express speed as a fraction of a maximal reference, the underlying constructs represent fundamentally different physiological and mechanical capacities. As such, it is vital that the denominator variable is clearly described to avoid conceptual and subsequent methodological misinterpretations. The final speed attained in an intermittent field test should be interpreted as a composite performance outcome influenced by aerobic fitness, anaerobic capacity, running economy, and acceleration-deceleration ability (21–23). In contrast, MS derived from a 40-m linear sprint test would be primarily determined by sprint mechanical factors, such as horizontal force production, the effectiveness of force application, stride characteristics, and neuromuscular function (24–26). Consequently, prescribing the same relative intensity (e.g., 75% MS or 75% Final Speed) on the basis of these different tests does not necessarily impose equivalent physiological or mechanical loads across athletes. Although the present paper focuses on terminology, practitioners should recognize that the validity of normalized thresholds, meaning scaled according to a player's MS, also depends on the procedures used to determine MS.

## 4 Concluding remarks

From a methodological perspective, studies analyzing running demands in soccer should clearly report how MS was obtained, whether thresholds were absolute or derived from individual player data, and how normalized intensities were calculated. Establishing clearer conceptual distinctions between these approaches would reduce ambiguity and facilitate comparisons across studies. In particular, it is important to distinguish between absolute thresholds based on fixed speed values, relative comparisons that contextualize performance against external references, individualized thresholds derived from player-specific data, and normalized intensities that mathematically scale running speeds relative to a physiological or biomechanical reference.

Given the substantial inter-individual variability in MS among soccer players, expressing running speed as a percentage of MS may offer a conceptually coherent framework when locomotor demands are intended to be interpreted relative to each player's maximal sprinting characteristic. Through normalization, running intensity can be contextualized relative to individual performance limits, which may enhance the interpretation of external-load exposure and load–response relationships. However, this should not be interpreted as evidence that normalized thresholds are inherently superior to alternative approaches, as the suitability of a given method ultimately depends on the specific research question and practical objective. Absolute and externally referenced thresholds may remain useful for comparisons between players, positions, teams, or competitive levels, whereas normalized thresholds may be particularly relevant when the purpose is to contextualize running demands relative to individual sprinting characteristics. Accordingly, each approach should be applied consistently with its methodological definition and intended purpose, while avoiding unnecessary terminological overlap.

## Data Availability

The original contributions presented in the study are included in the article; further inquiries can be directed to the corresponding author.

## References

[1] Oliva-Lozano JM Martín-Fuentes I Granero-Gil P Muyor JM. Monitoring elite soccer players physical performance using real-time data generated by electronic performance and tracking systems. J Strength Cond Res. (2022) 36(11):3224–8. 10.1519/JSC.0000000000004082 34175882

[2] Robertson S Duthie GM Ball K Spencer B Serpiello FR Haycraft J. Challenges and considerations in determining the quality of electronic performance & tracking systems for team sports. Front Sports Act Living. (2023) 5:1266522. 10.3389/fspor.2023.1266522 38173696 PMC10761404

[3] Pimenta R Antunes H Ribeiro J Nakamura FY. Should GPS data be normalized for performance and fatigue monitoring in soccer? A theoretical–practical discussion on sprinting. Ger J Exerc Sport Res. (2025). Available online at: https://link.springer.com/10.1007/s12662-025-01048-7 (Accessed March 10, 2026). 40636886 10.3389/fspor.2025.1603767 PMC12239930

[4] Pimenta R Antunes H Ribeiro J Nakamura FY. Should GPS data be normalized for performance and fatigue monitoring in soccer? A theoretical-practical discussion on high-speed running. Front Sports Act Living. (2025) 7:1603767. 10.3389/fspor.2025.1603767 40636886 PMC12239930

[5] Silva H Nakamura FY Loturco I Ribeiro J Marcelino R. Analyzing soccer match sprint distances: a comparison of GPS-based absolute and relative thresholds. Biol Sport. (2024) 41(3):223–30. 10.5114/biolsport.2024.133663 38952912 PMC11167479

[6] Gualtieri A Rampinini E Dello Iacono A Beato M. High-speed running and sprinting in professional adult soccer: current thresholds definition, match demands and training strategies. A systematic review. Front Sports Act Living. (2023) 5:1116293. 10.3389/fspor.2023.1116293 36860737 PMC9968809

[7] Carling C Bradley P McCall A Dupont G. Match-to-match variability in high-speed running activity in a professional soccer team. J Sports Sci. (2016) 34(24):2215–23. 10.1080/02640414.2016.1176228 27144879

[8] Clemente F Ramirez-Campillo R Beato M Moran J Kawczynski A Makar P. Arbitrary absolute vs. Individualized running speed thresholds in team sports: a scoping review with evidence gap map. Biol Sport. (2023) 40(3):919–43. 10.5114/biolsport.2023.122480 37398971 PMC10286616

[9] Abt G Lovell R. The use of individualized speed and intensity thresholds for determining the distance run at high-intensity in professional soccer. J Sports Sci. (2009) 27(9):893–8. 10.1080/02640410902998239 19629838

[10] Padrón-Cabo A Solleiro-Duran D Lorenzo-Martínez M Nakamura FY Campos-Vázquez MÁ Rey E. Application of arbitrary and individualized load quantification strategies over the weekly microcycle in professional soccer players. Biol Sport. (2024) 41(1):153–61. 10.5114/biolsport.2024.129481 PMC10765452 38188102

[11] Rago V Brito J Figueiredo P Krustrup P Rebelo A. Application of individualized speed zones to quantify external training load in professional soccer. J Hum Kinet. (2020) 72:279–89. 10.2478/hukin-2019-0113 32269668 PMC7126260

[12] Pimenta R Antunes H Maia F Ribeiro J Nakamura FY. Sprint and high-speed running in soccer: Should we use absolute or normalized thresholds? J Hum Kinet. (2025). Available online at: https://jhk.termedia.pl/Sprint-and-High-Speed-Running-in-Soccer-Should-We-Use-Absolute-or-Normalized-Thresholds,209540,0,2.html (Accessed March 10, 2026).

[13] Castellano J Blanco-Villaseñor A Alvarez D. Contextual variables and time-motion analysis in soccer. Int J Sports Med. (2011) 32(6):415–21. 10.1055/s-0031-1271771 21590641

[14] Martínez-Cabrera FI Núñez-Sánchez FJ Losada J Otero-Esquina C Sánchez H D Hoyo M. Use of individual relative thresholds to assess acceleration in young soccer players according to initial speed. J Strength Cond Res. (2021) 35(4):1110–8. 10.1519/JSC.0000000000002902 30507732

[15] Midgley AW McNaughton LR Jones AM. Training to enhance the physiological determinants of long-distance running performance: can valid recommendations be given to runners and coaches based on current scientific knowledge? Sports Med. (2007) 37(10):857–80. 10.2165/00007256-200737100-00003 17887811

[16] Achten J Jeukendrup AE. Heart rate monitoring: applications and limitations. Sports Med. (2003) 33(7):517–38. 10.2165/00007256-200333070-00004 12762827

[17] Suchomel TJ Nimphius S Stone MH. The importance of muscular strength in athletic performance. Sports Med. (2016) 46(10):1419–49. 10.1007/s40279-016-0486-0 26838985

[18] Jaric S. Muscle strength testing: use of normalisation for body size. Sports Med. (2002) 32(10):615–31. 10.2165/00007256-200232100-00002 12141882

[19] Pimenta R Maia F Silva H Nakamura FY. The speed dynamics of different sprint and acceleration exercises applied during football training. Sci Rep. (2025) 15(1):29543. 10.1038/s41598-025-04641-w 40796906 PMC12343760

[20] Ammann L Altmann S Ruf L Sperlich B. Seasonal analysis of match load in professional soccer players: an observational cohort study of a Swiss U18, U21 and first team. Front Physiol. (2022) 13:1023378. 10.3389/fphys.2022.1023378 36685210 PMC9846105

[21] Silva AF Alvurdu S Akyildiz Z Clemente FM. Relationships of final velocity at 30-15 intermittent fitness test and anaerobic speed reserve with body composition, sprinting, change-of-direction and vertical jumping performances: a cross-sectional study in youth soccer players. Biology (Basel). (2022) 11(2):197. 10.3390/biology11020197 35205064 PMC8869283

[22] Scott BR Hodson JA Govus AD Dascombe BJ. The 30-15 intermittent fitness test: can it predict outcomes in field tests of anaerobic performance? J Strength Cond Res. (2017) 31(10):2825–31. 10.1519/JSC.0000000000001563 27442337

[23] Ghazzagh A Niknam A Shahrbanian S. Validity and agreement of the 30-15 intermittent fitness test for estimating aerobic and performance-related parameters: a systematic review and meta-analysis. BMC Sports Sci Med Rehabil. (2026) 18:228. 10.1186/s13102-026-01616-w 41896967 PMC13147874

[24] Haugen TA Breitschädel F Seiler S. Sprint mechanical variables in elite athletes: are force-velocity profiles sport specific or individual? PLoS One. (2019) 14(7):e0215551. 10.1371/journal.pone.0215551 31339890 PMC6655540

[25] Buchheit M Samozino P Glynn JA Michael BS Al Haddad H Mendez-Villanueva A. Mechanical determinants of acceleration and maximal sprinting speed in highly trained young soccer players. J Sports Sci. (2014) 32(20):1906–13. 10.1080/02640414.2014.965191 25356503

[26] Miyashiro K Nagahara R Yamamoto K Nishijima T. Kinematics of maximal speed sprinting with different running speed, leg length, and step characteristics. Front Sports Act Living. (2019) 1:37. 10.3389/fspor.2019.00037 33344960 PMC7739839

